# Why sample selection matters in exploratory factor analysis: implications for the 12-item World Health Organization Disability Assessment Schedule 2.0

**DOI:** 10.1186/s12874-017-0309-5

**Published:** 2017-03-11

**Authors:** Cadeyrn J. Gaskin, Sylvie D. Lambert, Steven J. Bowe, Liliana Orellana

**Affiliations:** 1 Biostatistics Unit, Faculty of Health, Deakin University, Locked Bag 20001, Geelong, VIC 3220 Australia; 2 Ingram School of Nursing, Faculty of Medicine, McGill University, Montreal, QC Canada; 3 St. Mary’s Research Centre, Montreal, QC Canada

**Keywords:** Exploratory factor analysis, Eligibility criteria, Sample selection, Instrument validation, Psychometric assessment, WHODAS 2.0, ICF, Disability

## Abstract

**Background:**

Sample selection can substantially affect the solutions generated using exploratory factor analysis. Validation studies of the 12-item World Health Organization (WHO) Disability Assessment Schedule 2.0 (WHODAS 2.0) have generally involved samples in which substantial proportions of people had no, or minimal, disability. With the WHODAS 2.0 oriented towards measuring disability across six life domains (cognition, mobility, self-care, getting along, life activities, and participation in society), performing factor analysis with samples of people with disability may be more appropriate. We determined the influence of the sampling strategy on (a) the number of factors extracted and (b) the factor structure of the WHODAS 2.0.

**Methods:**

Using data from adults aged 50+ from the six countries in Wave 1 of the WHO’s longitudinal Study on global AGEing and adult health (SAGE), we repeatedly selected samples (*n* = 750) using two strategies: (1) simple random sampling that reproduced nationally representative distributions of WHODAS 2.0 summary scores for each country (i.e., positively skewed distributions with many zero scores indicating the absence of disability), and (2) stratified random sampling with weights designed to obtain approximately symmetric distributions of summary scores for each country (i.e. predominantly including people with varying degrees of disability).

**Results:**

Samples with skewed distributions typically produced one-factor solutions, except for the two countries with the lowest percentages of zero scores, in which the majority of samples produced two factors. Samples with approximately symmetric distributions, generally produced two- or three-factor solutions. In the two-factor solutions, the *getting along* domain items loaded on one factor (commonly with a *cognition domain* item), with remaining items loading on a second factor. In the three-factor solutions, the *getting along* and *self-care* domain items loaded separately on two factors and three other domains (*mobility*, *life activities*, and *participation in society*) on the third factor; the *cognition* domain items did not load together on any factor.

**Conclusions:**

High percentages of participants with no disability (i.e., zero scores) produce heavily censored data (i.e., floor effects), limiting data heterogeneity and reducing the numbers of factors retained. The WHODAS 2.0 appears to have multiple closely-related factors. Samples of convenience and those collected for other purposes (e.g., general population surveys) would usually be inadequate for validating measures using exploratory factor analysis.

## Background

The influence of sample selection on factor analytic solutions has long been recognised [1–3]. Factor analysis performed on data from diverse populations (e.g., samples from the general population versus samples of people with specific characteristics) can generate different factor solutions, both in terms of the numbers of factors extracted and factor structures [2]. In the development [4, 5] and subsequent evaluation [6, 7] of the World Health Organization Disability Assessment Schedule 2.0 (WHODAS 2.0), general population samples have often been used for factor analyses. With the WHODAS 2.0 designed to measure functioning and disability in adults, the use of samples including people with and without disability makes sense. If, as the name suggests, the instrument is concerned with assessing disability, however, then using data from samples of people with disability to validate the instrument would seem more appropriate.

Selecting appropriate samples is a key consideration when planning to perform factor analysis [8–11]. Homogenous samples, for example, restrict variance and, therefore, reduce factor loadings [10]. Factor analytic outcomes for a measure of intelligence, for instance, would be quite different when study samples are drawn from the general population (broad range of possible scores) than for, say, samples of postgraduate students (comparatively narrow range of possible scores). For this reason, sampling strategies need to facilitate the selection of participants who are likely to exhibit the range of possible values of the characteristics of interest [8, 9]. Samples that have adequate representation of the heterogeneity inherent in the characteristic being measured are preferable [9–11]. There needs to be balance between people who would score high on a proposed scale and those who would score low [9]. Ensuring that people who are likely to have a diverse range of scores are well represented takes precedence over selecting a sample that is representative of some identified population. Despite this advice, however, there seems to be few examples in the literature of how sampling influences factor analytic outcomes [2].

The WHODAS 2.0 is a cross-cultural multidimensional measure of functioning and disability in major life domains [4, 5]. Full (36 item) and short (12 item) “screener” versions of the instrument have been developed, the latter of which is the focus of this paper. The WHODAS 2.0 was designed to operationalise the International Classification of Functioning, Disability and Health (ICF) [12], which positions functioning and disability on a continuum, rather than binary opposites. As such, the model and measure were both designed to apply to all people, not just those with disability. Given this perspective, it would seem logical to use samples from the general population for validation work, including factor analysis.

The problem is, however, that the WHODAS 2.0 items focus on the difficulties experienced (from none to extreme) in performing certain activities. That is, the measure assesses the degree of disability without adequately operationalizing functioning. The highest level of functioning a respondent can report is the mere absence of difficulties. There is broad variation in function among people who may report having no difficulties performing a given activity. Take, for example, the item asking about the degree of difficulty experienced in walking a long distance such as a kilometre. A person who is physically unfit, but unimpaired, and an elite marathon runner may both indicate no difficulty performing this activity, but there are clear differences in their levels of functioning. The scale, therefore, censors the responses of people who experience no difficulty with the activities listed in the WHODAS 2.0. Given that the WHODAS 2.0 seems to operationalise disability, and not functioning, assessing the measure’s validity with samples of people with disability would seem most appropriate.

In the initial psychometric work on the WHODAS 2.0, factor analysis was undertaken with the pooled data of samples from several populations (general population, people with physical problems, people with mental or emotional problems, people with problems related to alcohol and drugs) in 14 countries [5]. Pooling data from several populations for factor analysis can be problematic [13]. If there are notable differences in item means between samples, then the inter-correlations between items can be affected when data are pooled. Correlations found between items in pooled data may be due to differences between samples, and may not be present (or, at least, to the same magnitude) when samples are factor analysed separately. In the case of the WHODAS 2.0, there is a reasonable likelihood that item means differed for the various populations (e.g., people with physical problems may have been experiencing disability to a greater extent than the general population), which may have affected item inter-correlations.

Subsequent psychometric evaluations of the 12-item (screener) version of the WHODAS 2.0 have involved samples of adults [7], older adults [6], and people with Huntington disease [14]. In the first two of these studies (i.e., non-disability-specific populations), many of the samples had high percentages of zero scores (ranging up to 75.7% in urban China) on the WHODAS 2.0 [6, 7]. A zero score indicates no activity limitations nor participation restrictions (i.e., no disability). Of the 12 samples used in these two studies, exploratory factor analyses showed there to be a single factor in each of seven samples and two factors in each of five samples. In contrast to studies showing one- and two-factor solutions, Carlozzi et al. [14] used confirmatory factor analysis to demonstrate that a six-factor structure (in which the six factors were the six domains of the WHODAS 2.0) fitted their data well. In their sample of people with Huntington disease there were no zero scores (i.e., no floor effects), but 19.5% of the sample had the highest possible score on the WHODAS 2.0 (i.e., ceiling effects). The amount of censorship in this study (i.e., ceiling and floor effects) was much less than in studies involving samples of adults [7] and older adults [6]. Although these results are not directly comparable due to the methods of analysis used (i.e., exploratory versus confirmatory factor analysis), they support the premise that the criteria used to select participants can influence factor analytic outcomes.

The factor structure of the WHODAS 2.0 has practical implications for the way in which the instrument is scored. In its initial development, the WHODAS 2.0 was considered unidimensional, which prompted the development of a weighted scoring method based on item response theory [4, 5]. If the WHODAS 2.0 was found not to be unidimensional, the use of this scoring method would need to be reconsidered.

In this study, we investigated the ramifications of using general population (rather than disability-specific) samples on the generation of factor analytic solutions using the 12-item WHODAS 2.0. Using repeated sampling from population-based data, we assessed the influence of different sampling schemes on the number of factors extracted. We then investigated the factor structure of the WHODAS 2.0.

## Method

### Design

Wave 1 data from the World Health Organization’s longitudinal Study on global AGEing and adult health (SAGE) were used for this investigation. The SAGE survey involves nationally representative samples of people aged 50+ from six countries (China, Ghana, India, Mexico, Russian Federation, and South Africa) with smaller samples of people aged 18 to 49 (not included in the present study). A description of the study methods has been provided elsewhere [15]. Briefly, multistage cluster sampling strategies were employed, in which households were allocated to one of two categories: (1) 50+ households and (2) 18–49 households. For each household, one household questionnaire was completed. For the 50+ households, all individuals aged 50+ were invited to be interviewed. The interviews were conducted face-to-face using either paper-based or electronic questionnaires.

### World Health Organization disability assessment schedule 2.0

The “screener” version of the WHODAS 2.0 has 12 items, with 2 items from each of six domains (cognition, mobility, self-care, getting along, life activities, and participation in society [4, 5]). Participants are asked about how much difficulty they have had performing certain activities in the past 30 days, and respond to each item on a 5-point Likert scale anchored with *none* (0) and *extreme or cannot do* (4). Summary scores can be created using simple scoring (whereby responses to each item are summed) or complex scoring (in which items are weighted based on item response theory). Using simple scoring, summary scores can range from 0 to 48, with higher scores indicating greater disability. Initial work showed that the 12-item WHODAS 2.0 explained 81% of the variance of the full, 36-item version [4].

### Analytical approach

Because this study was focused on the psychometric properties of the WHODAS 2.0, no survey weights were used in the analysis and only participants with complete data for the 12 WHODAS 2.0 items were included. Summary scores for the WHODAS 2.0 were calculated using the simple scoring method (i.e., summing the responses for each item) [5].

For each country the following approach was used to determine the influence of the type of sample selected for exploratory factor analysis on the number of factors extracted. Random samples of 750 adults were repeatedly selected from the country data using two sampling strategies: (1) simple random sampling that reproduced the distribution of WHODAS 2.0 summary scores in the SAGE dataset (referred to as *skewed distributions* in this paper) and (2) stratified random sampling with selection probabilities designed to obtain an approximately symmetric distribution around the median value of the WHODAS 2.0 summary scores (referred to as *approximately symmetric distributions* in this paper). Although we would have preferred to have used a screening strategy to identify people with disability using data other than that provided using the WHODAS 2.0, no other measure in the SAGE survey was suitable for this purpose. For each strategy, 1,000 samples of 750 adults were obtained. Given our expectation that there would be few items loading on each factor and communalities were likely to be reasonably high (i.e., .60 to .80), the sample size of 750 adults was deemed appropriate [16]. For each one of the 1,000 samples, the number of factors to retain was determined using parallel analysis with polychoric correlations, with principal components analysis as the method of extraction and the mean eigenvalue criterion [16].

To investigate the factor structure of the WHODAS 2.0, exploratory factor analysis was performed on one random sample (*n* = 750) with a skewed distribution and one with an approximately symmetric distribution from each country. These samples were required to have the same number of factors as the majority of the 1,000 samples in our previous analysis. The Kaiser-Meyer-Olkin measures of sampling adequacy for the correlation matrix as a whole and for each item [17, 18] were used to determine whether the data were suitable for factor analysis. Values above .60 are considered necessary for factor analysis [19]. The number of factors in each sample was determined using parallel analysis with polychoric correlations, principal components analysis, and the mean eigenvalue criterion. To obtain the factor solutions, minimum residuals estimations with polychoric correlations was the extraction method and Geomin was used to rotate factors [16].

## Results

### Descriptive statistics

Data from 31,251 adults aged 50+ from the six countries who had responded to all 12 WHODAS items were included in the analysis (Table 1). The percentages of female participants ranged from 47%, in Ghana, to 64%, in Russian Federation. The mean average ages of participants ranged from 62 years, in India, to 68 years, in Mexico.

**Table 1:** Demographic characteristics of the participants and properties of the distributions of WHODAS 2.0 summary scores by country

	China	India	Mexico	Russian Federation	South Africa	Ghana
Sample size (*N*)^a^	12,226	6,095	2,041	3,305	3,461	4,123
Demographic characteristics						
Female sex	53%	49%	60%	64%	58%	47%
Age – mean (standard deviation)	63(9)	62(9)	68(9)	64(10)	63(10)	64(11)
WHODAS 2.0 Score Distribution^b^						
25th/50th/75th percentiles	0/2/5	5/10/16	2/5/12	3/6/12	0/5/13	2/8/15
Mean scores (standard deviation)	3.52(5.24)	11.43(8.61)	7.79(8.18)	8.28(7.74)	8.01(8.76)	9.46(8.56)
Skewness (standard error)	2.82(.02)	0.97(.03)	1.48(.05)	1.45(.04)	1.23(.04)	1.07(.04)
Percentage of participants with zero scores	32%	6%	16%	7%	26%	15%

*Note.* ^a^Only adults aged 50+ with no missing value in WHODAS items responses were included

^b^12-item WHODAS 2.0 summary scores (calculated as the sum of item scores) can range from 0 to 48, with higher scores indicating greater activity limitations and participation restrictions

The distributions of WHODAS 2.0 summary scores differed markedly between the six countries (Table 1), with data from China and India having the most and least skewed distributions, respectively. The percentages of participants with zero scores ranged from 6% (India) to 32% (China). Across countries, participants generally had the most difficulty with “walking long distances” and the least difficulty with “getting dressed” (Table 2).

**Table 2:** Percentage distributions of the WHODAS 2.0 items for each country

	China					India					Mexico				
Response Options	0	1	2	3	4	0	1	2	3	4	0	1	2	3	4
Concentrating on doing something for 10 min	83	13	3	1	0	43	31	17	8	2	63	22	11	2	1
Learning a new task	44	35	16	5	0	32	27	21	15	5	52	28	15	3	1
Standing for long periods	77	15	6	2	0	25	30	23	19	4	44	23	20	11	3
Walking a long distance such as a kilometre	71	17	8	3	1	29	24	20	20	8	45	17	16	12	11
Bathing/washing whole body	93	5	2	1	0	82	12	4	2	1	78	12	6	3	2
Getting dressed	95	4	1	0	0	85	10	3	2	1	73	15	8	3	2
Making or maintaining friendships	91	7	2	0	0	56	22	15	6	2	76	17	6	1	0
Dealing with strangers	77	12	6	4	1	47	24	15	10	4	71	19	8	2	0
Taking care of household responsibilities	80	14	4	1	0	38	30	19	9	4	62	21	11	4	2
Day to day work	89	8	3	1	0	54	25	13	6	2	61	21	12	4	2
Joining in community activities	84	11	3	1	0	49	28	14	7	3	67	18	9	3	3
Emotionally affected by health condition	77	17	5	1	0	33	36	20	9	2	53	24	17	6	1
	Russian Federation					South Africa					Ghana				
Response Options	0	1	2	3	4	0	1	2	3	4	0	1	2	3	4
Concentrating on doing something for 10 min	77	17	3	2	0	58	17	19	5	1	59	27	12	2	1
Learning a new task	48	30	14	7	1	48	19	23	8	1	42	29	23	6	1
Standing for long periods	33	43	13	9	2	48	18	20	11	3	35	25	22	13	5
Walking a long distance such as a kilometre	33	32	14	12	10	48	13	19	14	6	38	21	21	12	8
Bathing/washing whole body	75	18	4	3	1	87	7	4	1	1	82	11	5	2	1
Getting dressed	82	14	2	2	1	88	6	4	1	0	82	11	6	1	1
Making or maintaining friendships	76	17	6	1	0	71	14	10	4	1	64	18	12	5	1
Dealing with strangers	69	22	7	2	0	66	14	14	5	1	65	16	13	5	1
Taking care of household responsibilities	55	33	7	5	1	62	16	15	5	2	51	26	17	5	2
Day to day work	61	28	7	4	1	75	11	9	4	1	51	18	22	6	3
Joining in community activities	55	28	7	7	3	66	13	13	5	3	51	20	16	8	5
Emotionally affected by health condition	16	40	29	14	1	54	19	19	7	1	46	29	19	5	1

### Numbers of factors in repeated sampling by country

**Fig. 1:**
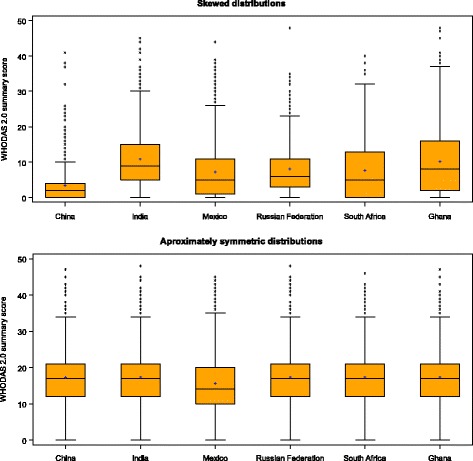
Box plots for the skewed and the approximately symmetric distributions of WHODAS 2.0 summary scores for each country

Figure 1 displays the distributions of the WHODAS summary scores in random samples from the skewed and approximately symmetric distributions, respectively. All of the samples analysed had one, two, or three factors. Differences between samples from the skewed and approximately symmetric distributions were found in the numbers of factors retained (Table 3). The skewed distributions of WHODAS 2.0 scores from each country revealed one factor for most samples of four countries (China, Mexico, South Africa, Ghana) and two factors for most samples of two countries (India, Russian Federation). Of the six countries, India and Russian Federation also had the lowest percentages of participants with zero scores (Table 1). The samples with approximately symmetric distributions, however, predominantly produced either two factors (China, India, Mexico, Russian Federation) or three factors (South Africa, Ghana).

**Table 3:** Number of samples in which one, two, and three factors were extracted for the *skewed distributions* (simple random sampling) and *approximately symmetric distributions* (stratified random sampling) for each country

	Skewed Distributions			Approximately Symmetric Distributions		
Number of Factors	1	2	3	1	2	3
China	993	7	0	0	1000	0
India	62	938	0	0	932	18
Mexico	894	106	0	0	1000	0
Russian Federation	6	994	0	0	1000	0
South Africa	1000	0	0	0	396	604
Ghana	587	413	0	0	0	1000

*Note. n* = 1000 samples of 750 adults per country per sampling approach

### Factor structure of samples with skewed and approximately symmetric distributions

The measures of sampling adequacy values for the correlation matrices (ranging from .81 to .93) and the individual variables (ranging from .61 to .97) indicated that the data were suitable for factor analysis. A consistent pattern of factor loadings was evident across sampling methods and countries when two factors were extracted (Tables 4 and 5). In all countries, two items in the domain *getting along* (“making new friends or maintaining current friendships”, “dealing with strangers”) loaded together strongly on one factor, commonly with a third item from the *cognition* domain (“learning a new task”). The other nine items loaded on the other factor.

**Table 4:** Factor pattern matrices for samples based on the *skewed distributions* of WHODAS 2.0 summary scores for each country

	China	India		Mexico	Russian Federation		South Africa	Ghana
	I	I	II	I	I	II	I	I
Concentrating on doing something for 10 min	**.74**	.**52**	-.32	**.76**	**.57**	.27	**.76**	**.80**
Learning a new task	**.59**	.12	**-.59**	**.62**	**.52**	.29	**.70**	**.70**
Standing for long periods	**.78**	**.74**	-.05	**.74**	**.81**	-.05	**.81**	**.73**
Walking a long distance such as a kilometre	**.82**	**.70**	-.04	**.75**	**.90**	-.09	**.79**	**.74**
Bathing/washing whole body	**.95**	**.86**	.08	**.88**	**.88**	.07	**.85**	**.83**
Getting dressed	**.95**	**.82**	.06	**.87**	**.90**	.03	**.89**	**.85**
Making or maintaining friendships	**.74**	.04	**-.86**	**.66**	.05	**.88**	**.72**	**.76**
Dealing with strangers	**.57**	-.06	**-.94**	**.61**	-.03	**.91**	**.66**	**.72**
Taking care of household responsibilities	**.82**	**.62**	-.15	**.86**	**.93**	-.02	**.89**	**.89**
Day to day work	**.91**	**.86**	.10	**.88**	**.94**	-.02	**.90**	**.74**
Joining in community activities	**.86**	**.54**	-.27	**.82**	**.77**	.11	**.88**	**.79**
Emotionally affected by health condition	**.70**	**.76**	-.08	**.70**	**.73**	-.01	**.80**	**.81**
Correlations between factors		I-II: −.55			I-II: .46			

*Note.* Matrices with one factor are unrotated solutions. Matrices with two factors were rotated using Geomin. Loadings ≥ .40 are bolded

**Table 5:** Factor pattern matrices for samples based on the *approximately symmetric distributions* of WHODAS 2.0 summary scores for each country

	China		India		Mexico		Russian Federation		South Africa			Ghana		
	I	II	I	II	I	II	I	II	I	II	III	I	II	III
Concentrating on doing something for 10 min	.38	.37	**.45**	-.25	**.43**	-.30	**.47**	.31	.37	.24	-.11	**.51**	.15	.13
Learning a new task	.06	**.44**	.18	**-.50**	.27	-.33	.32	.39	.33	.36	.10	.09	.38	.07
Standing for long periods	**.56**	-.02	**.68**	.04	**.68**	.16	**.66**	-.08	**.70**	-.07	-.02	.00	.21	**.70**
Walking a long distance such as a kilometre	**.68**	-.05	**.65**	.10	**.65**	.25	**.72**	-.11	**.78**	-.01	.11	-.06	.10	**.84**
Bathing/washing whole body	**.92**	-.01	**.78**	-.06	**.70**	-.21	**.86**	.07	.02	.05	**-.90**	**.88**	.05	-.06
Getting dressed	**.85**	.03	**.79**	-.04	**.74**	-.15	**.89**	.00	.03	.01	**-.98**	**1.02**	.05	-.15
Making or maintaining friendships	.14	**.80**	.02	**-.85**	.00	**-.90**	.01	**.89**	.01	**.87**	-.05	-.04	**.96**	-.06
Dealing with strangers	-.20	**.80**	-.08	**-.89**	.00	**-.84**	-.04	**.90**	-.05	**.79**	-.05	.01	**.96**	-.15
Taking care of household responsibilities	**.65**	.06	**.62**	-.04	**.69**	-.09	**.84**	.00	**.70**	-.03	-.23	.33	.26	**.48**
Day to day work	**.87**	-.05	**.80**	.14	**.87**	.09	**.88**	.01	**.50**	-.03	**-.49**	.39	-.06	**.51**
Joining in community activities	**.65**	.18	**.52**	-.25	**.58**	-.22	**.67**	.11	**.44**	.22	-.27	.00	**.46**	**.55**
Emotionally affected by health condition	**.48**	.12	**.67**	-.02	**.59**	.00	**.57**	.00	**.54**	.04	-.19	**.44**	-.07	**.49**
Correlations between factors	I-II: .46		I-II: −.33		I-II: −.32		I-II: .27		I-II: .27 I-III: −.49 II-III: −.33			I-II: .38 I-III: .33 II-III: .15		

*Note.* Matrices were rotated using Geomin. Loadings ≥ .40 are bolded

In South Africa and Ghana, for which three factors were obtained from samples with approximately symmetric distributions, the two *getting along* items loaded strongly on one same factor. The two *self-care* items (“bathing/washing your whole body”, “getting dressed”) loaded strongly on a second factor. The third factor included six items: the two *mobility* items (“standing for long periods”, “walking a long distance such as a kilometre”), the two *life activities* items (“taking care of your household responsibilities”, “your day to day work”), and the two *participation in society* items (“joining in community activities in the same way as anyone else can”, “emotionally affected by your health conditions”). The two *cognition items* (“concentrating on doing something for 10 min”, “learning a new task”) did not load highly on the same factor. One item (“your day to day work”) cross-loaded on two factors in the solution for South Africa, and three items (“your day to day work”, “joining in community activities in the same way as anyone else can”, “emotionally affected by your health conditions”) cross-loaded on two factors in the solution for Ghana.

The correlations between factors ranged in from |.15| to |.55|. When two factors were positively correlated, items loaded positively on each factor. When two factors were negatively correlated, items loaded positively on one factor and negatively on the other. In essence, therefore, all factors were positively correlated.

## Discussion

The findings illustrate that sample selection (in this instance, we mean the participant selection criteria combined with sampling methods) for a study in which exploratory factor analysis is planned can influence the factor solutions obtained. Consistent with findings from the initial [4, 5] and much of the subsequent [6, 7] psychometric work on the 12-item WHODAS 2.0, when we factor analysed data from samples of older adults from the SAGE study (i.e., the skewed distribution samples) we typically obtained one-factor, and sometimes two-factor, solutions. The choices of samples to use for these validation studies are problematic, however, because (a) the participant selection criteria were typically based on considerations other than validating the 12-item WHODAS 2.0 (e.g., obtaining information about mental health and wellbeing in the general population [7]), and (b) the sampling methods were not designed to achieve heterogeneity in the characteristic of interest.

Our findings are that different factor solutions (e.g., generally more factors) were generated from samples with approximately symmetric distributions on the WHODAS summary scores than those with skewed distributions. That is, sampling specifically designed to achieve heterogeneity with respect to disability resulted in more factors being retained than samples that included large proportions of people with no, or minimal, disability. These findings are consistent with previous research showing that when factor analyses are performed on data from different populations (e.g., a college sample versus a nationwide sample) different factor solutions are generated, both in terms of the numbers of factors extracted and factor structures [2]. Our results reinforce advice to obtain heterogeneous samples for factor analysis [9–11].

Limited heterogeneity has ramifications for the accuracy of factor analysis. Many zero scores – up to 75.7% in one sample [6] – means that large percentages of participants gave the same response for each of the 12 items of the WHODAS 2.0. For these participants, all items are perfectly correlated. In such large percentages, these responses tend to dominate the covariance matrix structure and obfuscate any latent factors. These findings are consistent with previous work showing that as Likert scale data become more skewed, methods for (a) determining the number of factors to rotate and (b) extracting factors are increasingly inaccurate [20–22].

The findings also serve to clarify what heterogeneity means when working with Likert scale data. Maximum heterogeneity in WHODAS 2.0 summary scores would involve including equal proportions of participants with each possible score (i.e., a uniform distribution). Such a sampling approach, however, would include meaningful proportions of people with summary scores at, or near, the extremes of the scale, which would create two issues. First, there are ceiling and floor effects. In the case of the WHODAS 2.0, we saw no evidence of a ceiling effect, but there was a substantial floor effect. Second, all items at, or near, either extreme are perfectly, or near perfectly, correlated, which affects the covariance matrix structure and the extraction of factors. This finding reiterates the importance of constructing measures in ways that avoid severe ceiling and floor effects in the populations for whom they have been primarily designed.

Our results do not support previously reported findings that the WHODAS 2.0 is unidimensional [5–7]. We speculate that the one-factor solutions found in previous research may be a consequence of sample selection rather than a reflection of the underlying dimensionality of this survey instrument. We are not the first, however, to conclude that more than one factor could be retained [6, 7, 14]. In studies involving adults [7] and older adults [6], two factors were present in some of the samples. Both teams of researchers preferred the one-factor solutions, however, on the basis of (a) results from methods of determining the number of factors to extract that have been shown to be inaccurate (e.g., the eigenvalues greater than one rule, scree plots), (b) evidence (from confirmatory factor analysis) that a second-order one factor solution with six first-order factors (i.e., two items loading on each of the major life domains) fitted the data reasonably well [7], (c) differences across samples with respect to which items loaded on each factor (i.e., meaning that the factors are not easily interpretable), (d) the dominance of the first factor (in terms of variance explained), and (e) the results from the Mokken scale analysis [6]. One observation that can be made from the results of these studies is that (as in our study) two-factor solutions tended to emerge from samples with fewer zero scores.

Given that two-factor solutions for the 12-item WHODAS 2.0 have not been easily interpretable, some researchers have suggested that the measure be considered unidimensional, with “item difficulty” explaining the loading of items on separate factors [6]. In our study, the three-factor solutions were more interpretable than two factor solutions. In the three-factor solutions, the two *getting along* items and the two *self-care* items loaded strongly on separate factors. Even so, the items of three domains (*mobility*, *life activities*, *participation in society*) loaded on one factor, and the loadings of the *cognition* items were inconsistent across samples from different countries. Although item difficulty may contribute to these findings, alternative explanations include: (a) the samples in our study had limited numbers of participants with severe disability (therefore, restricting the heterogeneity within our samples, even under approximately symmetric sampling), (b) too few items may have been chosen to represent each domain when reducing the WHODAS 2.0 from 36 to 12 items (only two items on a factor is potentially problematic [23–25]), and (c) some of the items may not adequately represent the constructs underlying each domain (e.g., the item “emotionally affected by health condition” is meant to represent the *participation in society* domain, but tended to cross-load in solutions where three factors were obtained). Perhaps more factors would have been recovered (possibly aligning with the six domains) if we were able to include people with more severe impairments in our samples. The inconsistency within these findings make the unidimensional model for the WHODAS 2.0 easier for researchers to use.

Our findings, and those of previous studies [5–7], are probably due to the way the WHODAS 2.0 was designed. An instrument designed to measure functioning and disability in the general population needs a scale that can measure differences in the severity of disability as well as in the degree of functioning. The WHODAS 2.0, however, only measures severity of disability. Operationalising functioning as the absence of disability sharply censors the broad range of differences in human functioning. This mismatch between model and its operationalisation invites several possible remedies, including (a) reconceptualising the ICF to focus on disability, and (b) redeveloping the WHODAS 2.0 to measure broad differences in functioning, as well as disability. The first of these alternatives is rather regressive, with contemporary writings challenging the binary thinking on disability and normalcy [26, 27]. Producing a new measure of functioning and disability would seem the preferable option.

There are some limitations of this study. First, although the SAGE datasets available for each country were large, they contained few adults with severe disability, which reduced the potential heterogeneity we could achieve when sampling from the datasets. Second, we had no way of sampling people with disability other than through using the WHODAS 2.0 summary scores. The SAGE survey contains questions on a limited range of health conditions (e.g., arthritis, angina, chronic lung disease, depression). Therefore, other than using the WHODAS 2.0, there was no way of identifying people with disability in the dataset. Third, our study focused on adults aged 50+. Many of the participants could be expected to have acquired impairments later in life, which may mean their experiences of disability were different to, say, younger people with childhood-onset impairments. Therefore, our findings do not necessarily extend to younger people.

## Conclusions

Several messages from the findings of this study are worth emphasising. First, participant selection criteria and sampling methods matter when it comes to using exploratory factor analysis in the process of validating a measure. Samples that are not relevant to the focus of the measure (e.g., using population-based samples when disability-specific samples would be more appropriate) have the potential to generate heavily censored and highly skewed data, which are not usually sufficiently heterogeneous with respect to the characteristic of interest. Second, measures that produce severe ceiling or floor effects are problematic for exploratory factor analysis. Taking these first two points together, the findings highlight that the factors produced are a property of the measure and the population of interest. Third, the WHODAS 2.0 would not appear to be unidimensional. Rather, the instrument seems to incorporate several closely-aligned constructs. Therefore, we caution against following the recommendation to weight items [4, 5]. Using simple scoring (summing the responses for each item) would seem the safer option. We hope this work stimulates improvements in the use of factor analysis in validation exercises and encourages debate about how the ICF should be operationalised.

## Abbreviations

ICF: International Classification of Functioning, Disability and Health
SAGE: Study on global AGEing and adult health
WHO: World Health Organization
WHODAS 2.0: World Health Organization Disability Assessment Schedule 2.0

## References

[1] Thurstone LL (1945). The effects of selection in factor analysis. Psychometrika.

[2] Simón A (1979). Effects of selective sampling on a factor analysis. J Gen Psychol.

[3] Comrey AL (1978). Common methodological problems in factor analytic studies. J Consult Clin Psychol.

[4] Üstün TB, Chatterji S, Kostanjsek N, Rehm J, Kennedy C, Epping-Jordan J, Saxena S, Korff M, Pull C (2010). Developing the World Health Organization disability assessment schedule 2.0.. Bull World Health Organ.

[5] Üstün TB, Kostanjsek N, Chatterji S, Rehm J (2010). Measuring health and disability: manual for WHO disability assessment schedule (WHODAS 2.0).

[6] Sousa RM, Dewey ME, Acosta D, Jotheeswaran AT, Castro-Costa E, Ferri CP, Guerra M, Huang Y, Jacob KS, Pichardo JGR (2010). Measuring disability across cultures — the psychometric properties of the WHODAS II in older people from seven low- and middle-income countries. The 10/66 Dementia Research Group population-based survey. Int J Method Psychiatr Res.

[7] Andrews G, Kemp A, Sunderland M, Von Korff M, Ustun TB (2009). Normative data for the 12 item WHO disability assessment schedule 2.0. PLoS ONE.

[8] Cattell RB (1973). Personality and mood by questionnaire: a handbook of interpretive theory, psychometrics, and practical procedures.

[9] Gorsuch RL (1997). Exploratory factor analysis: its role in item analysis. J Pers Assess.

[10] Kline P (1994). An easy guide to factor analysis.

[11] Reise SP, Waller NG, Comrey AL (2000). Factor analysis and scale revision. Psychol Assess.

[12] World Health Organization (2002). Towards a common language for functioning, disability and health: ICF.

[13] Guilford JP (1952). When not to factor analyze. Psychol Bull.

[14] Carlozzi NE, Kratz AL, Downing NR, Goodnight S, Miner JA, Migliore N, Paulsen JS (2015). Validity of the 12-item World Health Organization disability assessment schedule 2.0 (WHODAS 2.0) in individuals with Huntington disease (HD). Qual Life Res.

[15] Kowal P, Chatterji S, Naidoo N, Biritwum R, Fan W, Lopez Ridaura R, Maximova T, Arokiasamy P, Phaswana-Mafuya N, Williams S (2012). Data resource profile: the World Health Organization study on global AGEing and adult health (SAGE). Int J Epidemiol.

[16] Gaskin CJ, Happell B (2014). On exploratory factor analysis: a review of recent evidence, an assessment of current practice, and recommendations for future use. Int J Nurs Stud.

[17] Kaiser HF (1970). A second generation little jiffy. Psychometrika.

[18] Kaiser HF, Rice J (1974). Little Jiffy, mark IV. Educ Psychol Meas.

[19] Tabachnick BG, Fidell LS (2001). Using multivariate statistics.

[20] Garrido LE, Abad FJ, Ponsoda V (2013). A new look at Horn’s parallel analysis with ordinal variables. Psychol Methods.

[21] Muthén B, Kaplan D (1992). A comparison of some methodologies for the factor analysis of non-normal Likert variables: a note on the size of the model. Br J Math Stat Psychol.

[22] Muthén B, Kaplan D (1985). A comparison of some methodologies for the factor analysis of non-normal Likert variables. Br J Math Stat Psychol.

[23] Marsh HW, Hau K-T, Balla JR, Grayson D (1998). Is more ever too much? The number of indicators per factor in confirmatory factor analysis. Multivariate Behav Res.

[24] Ding L, Velicer WF, Harlow LL (1995). Effects of estimation methods, number of indicators per factor, and improper solutions on structural equation modeling fit indices. Struct Equ Modeling.

[25] Little TD, Lindenberger U, Nesselroade JR (1999). On selecting indicators for multivariate measurement and modeling with latent variables: when “good” indicators are bad and “bad” indicators are good. Psychol Methods.

[26] Corker M, Shakespeare T, Shakespeare T, Corker M (2002). Mapping the terrain. Disability/postmodernity: embodying disability theory.

[27] Gaskin CJ (2015). On the potential for psychological researchers and psychologists to promote the social inclusion of people with disability: a review. Aust Psychol.

